# Mesenchymal Stromal Cell-Derived Factors Promote Tissue Repair in a Small-for-Size Ischemic Liver Model but Do Not Protect against Early Effects of Ischemia and Reperfusion Injury

**DOI:** 10.1155/2015/202975

**Published:** 2015-08-24

**Authors:** Suomi M. G. Fouraschen, Joshua H. Wolf, Luc J. W. van der Laan, Petra E. de Ruiter, Wayne W. Hancock, Job P. van Kooten, Monique M. A. Verstegen, Kim M. Olthoff, Jeroen de Jonge

**Affiliations:** ^1^Department of Surgery, Erasmus MC-University Medical Center, 3015 CE Rotterdam, Netherlands; ^2^Department of Surgery, Penn Transplant Institute, University of Pennsylvania, Philadelphia, PA 19104, USA; ^3^Department of Transplant Immunology, Pathology and Laboratory Medicine, The Children's Hospital of Philadelphia, Philadelphia, PA 19104-4318, USA

## Abstract

Loss of liver mass and ischemia/reperfusion injury (IRI) are major contributors to postresectional liver failure and small-for-size syndrome. Mesenchymal stromal cell- (MSC-) secreted factors are described to stimulate regeneration after partial hepatectomy. This study investigates if liver-derived MSC-secreted factors also promote liver regeneration after resection in the presence of IRI. C57BL/6 mice underwent IRI of 70% of their liver mass, alone or combined with 50% partial hepatectomy (PH). Mice were treated with MSC-conditioned medium (MSC-CM) or unconditioned medium (UM) and sacrificed after 6 or 24 hours (IRI group) or after 48 hours (IRI + PH group). Blood and liver tissue were analyzed for tissue injury, hepatocyte proliferation, and gene expression. In the IRI alone model, serum ALT and AST levels, hepatic tissue damage, and inflammatory cytokine gene expression showed no significant differences between both treatment groups. In the IRI + PH model, significant reduction in hepatic tissue damage as well as a significant increase in hepatocyte proliferation was observed after MSC-CM treatment. *Conclusion*. Mesenchymal stromal cell-derived factors promote tissue regeneration of small-for-size livers exposed to ischemic conditions but do not protect against early ischemia and reperfusion injury itself. MSC-derived factors therefore represent a promising treatment strategy for small-for-size syndrome and postresectional liver failure.

## 1. Introduction

Advances in surgical techniques have enabled large liver resections as well as split and living donor liver transplantation (LDLT). Transplantation of partial (living donor) liver grafts was introduced to help overcome donor organ scarcity and reduce waitlist mortality. Living donors undergo resection of approximately 40–60% of their liver volume, which is transplanted into the recipient. Without the exceptional capacity of the liver to regenerate and thereby compensate for tissue loss and restore homeostasis, these extensive resections and partial graft transplantations would not be possible [1–3]. Nevertheless, in case of adult to adult living donor liver transplantation both donors and partial graft recipients end up with a small-for-size liver, which is still associated with significant morbidity and even mortality [2, 4, 5]. In an attempt to decrease donor risk, smaller grafts (such as the left lobe of the liver) can be used, but this is limited by the increased risk of the recipient to develop small-for-size syndrome [6].

In these settings, both loss of a substantial part of the liver mass and the inevitable ischemia and reperfusion injury (IRI) are major mechanisms of hepatic injury [7, 8]. Effective therapeutic strategies to protect against IRI, enhance regeneration, and stimulate recovery could minimize donor and recipient risk.

A promising new therapeutic intervention can be found in mesenchymal stromal/stem cell (MSC) based strategies [9–12]. At the early beginning of research on MSCs they were thought only to provide a supportive niche for hematopoietic stem cells in the bone marrow. Meantime, they have been reported to reside in multiple tissue compartments, including lung, liver, and adipose tissue [13–15]. Our group showed that the adult human liver harbors a population of MSCs, highly similar to bone marrow MSCs in their multilineage differentiation potential as well as their genome-wide gene expression profiles, which is mobilized from liver grafts at time of transplantation [14, 16]. These liver-derived MSCs (L-MSCs) can be retrieved from the organ preservation solution and appear to have immunosuppressive capacities as well as multilineage differentiation potential [17]. Furthermore, we have reported that the trophic factors secreted by these L-MSCs stimulate liver regeneration after surgical resection, mainly by promoting hepatocyte proliferation and altering expression levels of regeneration-related genes [18, 19].

Previous research revealed that MSC-secreted trophic factors include a broad spectrum of growth factors, chemokines, and cytokines related to cellular growth and proliferation as well as immunomodulation in the setting of toxic liver injury and hepatic failure [20, 21]. In those experiments, a significant survival benefit with decreased signs of liver injury and increased hepatocyte proliferation was gained with conditioned medium, enriched mainly for IGFBP-1, leptin, and CCL2, but also containing IL-6 and TNF-*α*.

The use of these MSC-secreted factors in a clinical setting may have important advantages over the use of MSCs: there is no risk of rejection or possible malignant transformation and the factors can be produced ready-to-use in large clinical grade quantities [22]. The aim of this study is to investigate whether L-MSC-secreted factors are as effective to ameliorate hepatic IRI as well as to promote regeneration in a clinically relevant model of combined IRI and partial liver resection.

## 2. Materials and Methods

### 2.1. Animals

Male C57BL/6 mice (age 7–10 weeks) were obtained from Jackson Laboratories (Bar Harbor, ME, USA) and maintained in the animal facility on a 12/12 hour light/dark schedule. The animals had free access to food and drinking water. All animal experiments were performed with approval of the institutional animal welfare committee.

### 2.2. Human L-MSC Cultures and Conditioned Medium

Liver-derived MSCs were obtained from the UW organ preservation solution (Viaspan, Bristol-Myers Squibb, Woerden, Netherlands), collected after cold storage of human liver grafts for transplantations performed at the Erasmus Medical Center, Rotterdam, Netherlands. The Medical Ethical Council of the Erasmus Medical Center and the Institutional Biological Safety Committee of the Children's Hospital of Philadelphia approved the use of human donor material for medical research.

Mononuclear cells were isolated from the collected preservation fluids by density gradient centrifugation using Ficoll-Paque Plus (GE Healthcare, Uppsala, Sweden) and put into culture as previously described [14, 19]. Culture medium consisted of MEM alpha (Invitrogen, Grand Island, NY, USA) supplemented with 15% fetal bovine serum (Sigma-Aldrich, St. Louis, MO, USA), L-glutamine (Invitrogen), and penicillin and streptomycin (Invitrogen). The last three days before collecting the supernatant, L-MSCs were cultured under serum-free conditions. Culture medium was therefore changed to MEM alpha supplemented with 0.05% bovine serum albumin (Sigma-Aldrich), L-glutamine, penicillin, and streptomycin. The MSC-conditioned culture medium (MSC-CM) was collected three days after medium change of L-MSC cultures from passage 6–10. MSC-CM was concentrated approximately 25-fold by filtration with 3-kD molecular cut-off filters (Amicon Ultra, Millipore, Carrigtwohill, Ireland).

### 2.3. Surgical Procedures and MSC-CM Treatment

C57BL/6 mice were anesthetized and injected intraperitoneally with 100 U/kg heparin. After a midline laparotomy, ischemia and reperfusion injury (IRI) with or without partial hepatectomy (PH) was induced. All procedures were performed under clean conditions.

In the IRI alone group, 90 minutes of ischemic injury of 70% of the liver was induced by clamping the blood supply to the left lateral and median lobes with microvascular clamps. In this way the right lateral and caudate lobes served as a portacaval shunt, allowing survival of the animals during the ischemic period.

In the combined (IRI + PH) group, 60 minutes of ischemic injury was induced as described above, after which the right part of the median lobe, the right lateral lobe, and the caudate lobes were ligated and resected, leaving approximately 50% ischemic liver tissue. The combination of ischemic injury with 50% hepatectomy did not allow an ischemic period of more than 60 minutes, without affecting survival. During the ischemic period in both groups, the abdominal cavity was covered with saline-moistened gauzes and the animals were kept under anesthesia on a warming plate to conserve body temperature. At the end of the surgical procedures the peritoneum and skin were sutured separately.

In both groups, half of the animals were treated with 200 *μ*L of the concentrated serum-free MSC-CM, injected intraperitoneally at the end of the surgical procedure. The other animals were treated similarly with concentrated serum-free unconditioned medium (UM). This unconditioned medium consisted of culture medium treated exactly the same as the serum-free MSC-CM but without the presence of L-MSCs. The animals in the IRI alone group were sacrificed either 6 or 24 hours after surgery, based on existing literature on liver IRI, which shows clearest signs of tissue damage and highest levels of serum injury markers at these time points. The animals in the IRI + PH group were treated a second time with MSC-CM or UM after 24 hours and were sacrificed 48 hours after surgery, as this was the time point showing largest effects on hepatocyte proliferation in our previous study [18]. From all animals (*n* = 8 per study group) blood and liver tissue were collected to further investigate the effects of MSC-CM on serum markers of liver function, tissue injury, hepatocyte proliferation, and hepatic gene expression in the early phase after liver injury.

### 2.4. Weight Calculations

Animals were weighed daily prior to treatment. In the IRI + PH group the resected liver mass was weighed after PH. The initial total liver weight was calculated as follows:

(1) $$\begin{array}{c} \frac{\text{Resected liver weight}}{50}*100\;\left(\;g\;\right). \end{array}$$

At time of sacrifice the animals, and in the IRI + PH group also their regenerated liver mass, were weighed. The percentage of reconstitution of the liver was calculated by

(2) $$\begin{array}{c} \frac{\text{Regenerated liver weight}}{\text{Initial total liver weight}}*100\left(\;\%\;\right). \end{array}$$

The liver to body weight ratio was calculated by

(3) $$\begin{array}{c} \frac{\text{Regenerated liver weight}}{\text{Body weight at time of harvest}}*100\left(\;\%\;\right). \end{array}$$

### 2.5. Immunohistochemistry

BrdU staining (5-bromo-2′-deoxyuridine is incorporated in the DNA of proliferating cells) was used to investigate the number of proliferating hepatocytes. One hour prior to sacrifice, animals were injected intraperitoneally with 50 mg/kg BrdU (B5002, Sigma-Aldrich). Liver tissue was harvested and processed to 4 mm thick formalin fixed, paraffin embedded sections. They were stained for BrdU using the following staining protocol: antigen retrieval was achieved by boiling the sections in 0.01 M sodium citrate, pH 6.0 (microwave 1000 Watt; 1 × 7 and 2 × 3 minutes). Endogenous peroxidase was blocked by 0.6% H_2_O_2_ in PBS for 30 minutes at room temperature, after which DNA was denatured by incubation for 1 hour at 37°C in 0.1 M HCl in aqua dest. Aspecific binding was prevented by 0.5% milk powder supplemented with 0.15% glycin in PBS (blocking buffer). Sections were incubated overnight at 4°C with monoclonal mouse anti-BrdU (Bu20a; DakoCytomation, Glostrup, Denmark; 1 : 80 in blocking buffer). The next day sections were incubated for 30 minutes at room temperature with polyclonal rabbit anti-mouse IgG/HRP (P0161; DakoCytomation; 1 : 1000 in blocking buffer). After antibody incubation sections were incubated with DAB-solution and counterstained with hematoxylin. Per animal 4 high power fields (HPF; 400x) were analyzed for BrdU positive hepatocytes. Liver tissue sections were also stained with hematoxylin and eosin (H&E) using a standard staining protocol.

### 2.6. Serum Analysis of Transaminase Levels

Blood samples were collected at time of sacrifice in heparin coated microtubes. After collection, samples were centrifuged (19 minutes, 1800 rpm) to separate the serum, which was then further analyzed at the clinical chemical core facility of The Children's Hospital of Philadelphia to determine alanine aminotransferase (ALT) and aspartate aminotransferase (AST) levels.

### 2.7. Real-Time Quantitative RT-PCR

At time of sacrifice, liver tissue was stored overnight at 4°C and thereafter at −80°C in Allprotect Tissue Reagent (Qiagen, Valencia, CA, USA) for RNA preservation. Total RNA was extracted using Trizol (Qiagen) and chloroform after mechanical disruption of the tissue. RNA was precipitated in 75% ethanol and dissolved in RNase-free water. RNA quantity and quality were analyzed using a NanoDrop ND-1000 (Thermo Fisher Scientific, Wilmington, DE, USA). One microgram of RNA was reverse-transcribed to cDNA using an iScript cDNA Synthesis Kit (Bio-Rad Laboratories, Hercules, CA, USA). PCR primers (presented in Table 1) were synthesized by Isogen Life Science (Maarssen, Netherlands) and Biolegio (Nijmegen, Netherlands). Real-time quantitative RT-PCR was performed with a SensiMix SYBR & Fluorescein Kit (Bioline, London, United Kingdom) and MyIQ real-time PCR detection system (Bio-Rad Laboratories) according to the manufacturer's instruction.

### 2.8. MSC-CM Mass Spectrometry Analysis

Twenty-five-fold concentrated serum-free MSC-CM (50-kD molecular cut-off filters; Amicon Ultra) was used for protein analysis to elucidate effects of MSC-CM on IRI and hepatic proliferation. One-dimension SDS-PAGE gel lanes were cut into 2-mm slices using an automatic gel slicer and subjected to in-gel reduction with dithiothreitol, alkylation with iodoacetamide, and digestion with trypsin (sequencing grade; Promega, Leiden, Netherlands), essentially as described by Wilm et al. [23]. Nanoflow LC-MS/MS was performed on 1100 series capillary liquid chromatography system (Agilent Technologies, Amstelveen, Netherlands) coupled to an LTQ-Orbitrap mass spectrometer (Thermo Scientific, USA) operating in positive mode and equipped with a nanospray source. Peptide mixtures were trapped on a ReproSil C18 reversed phase column (column dimensions 1.5 cm × 100 *μ*m, packed in-house; Dr. Maisch GmbH, Ammerbuch, Entringen, Germany) at a flow rate of 8 *μ*L/min. Peptide separation was performed on ReproSil C18 reversed phase column (column dimensions 15 cm × 50 *μ*m, packed in-house; Dr. Maisch GmbH) using a linear gradient from 0 to 80% B (A = 0.1% formic acid; B = 80% (v/v) acetonitrile, 0.1% formic acid) in 70 min and at a constant flow rate of 200 nL/min using a splitter.

The column eluent was directly sprayed into the electrospray ionization source of the mass spectrometer. Mass spectra were acquired in continuum mode; fragmentation of the peptides was performed in data-dependent mode. Peak lists were automatically created from raw data files using the Mascot Distiller software (version 2.3; MatrixScience, London, UK). The Mascot search algorithm (version 2.2; MatrixScience) was used for searching against a customized database containing all IPI_human protein sequences (release 2010_09). The peptide tolerance was typically set to 10 ppm and the fragment ion tolerance was set to 0.8 Da. A maximum number of 2 missed cleavages by trypsin were allowed and carbamidomethylated cysteine and oxidized methionine were set as fixed and variable modifications, respectively. The Mascot score cut-off value for a positive protein hit was set to 65. Individual peptide MS/MS spectra with Mascot scores below 40 were checked manually and either interpreted as valid identifications or discarded. Functional analyses of these peptides were performed using Ingenuity Pathway Analysis (Ingenuity Systems, Redwood City, CA, USA).

### 2.9. Statistical Analysis

All data are presented as mean ± SEM and statistical analyses were performed using the Mann-Whitney test with GraphPad Prism software and *p* ≤ 0.05 was considered statistically significant.

## 3. Results

### 3.1. Body and Liver Weight after IRI with or without PH Are Not Affected by MSC-CM

**Figure 1:**
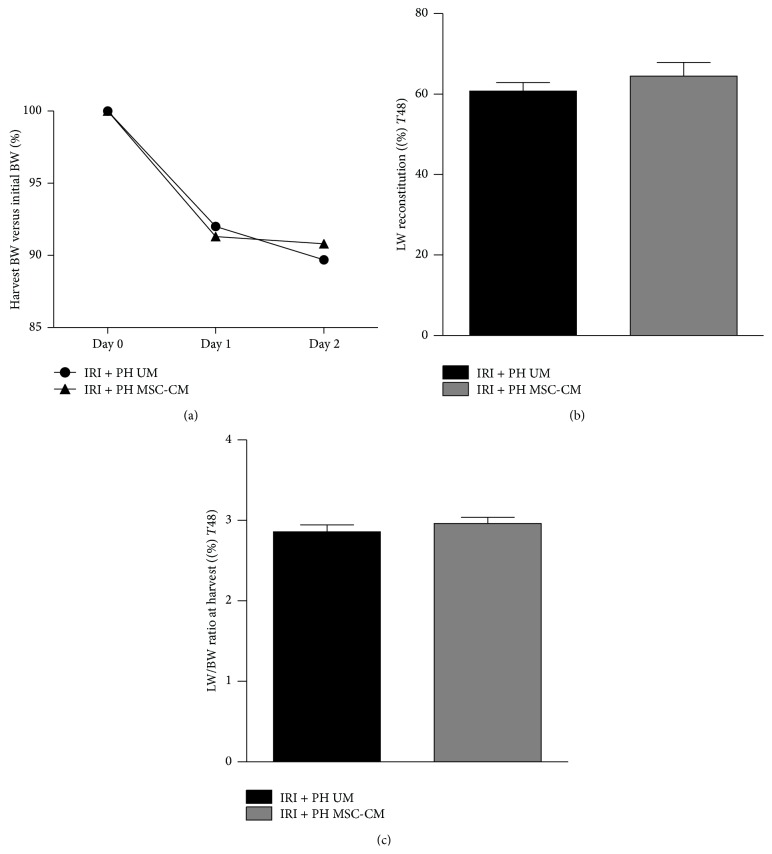
Effects of MSC-CM on body and liver weight after IRI + PH. (a) Body weight change from surgery to harvest; (b) harvest liver weight versus initial liver weight; (c) harvest liver weight to body weight ratio.

In the IRI alone group, no significant differences in body weight change were observed (data not shown). In the IRI + PH group, all animals showed a decrease in body weight on postoperative days 1 and 2, but without statistically significant differences between the MSC-CM and UM treated groups (9.2% versus 10.3% decrease of initial body weight, *p* = 0.96; Figure 1(a)). Liver weight after PH increased with 29.0% in the MSC-CM treated group (from 50% to 64.5% of the initial liver weight) and with 21.6% in the UM group (from 50% to 60.8%, *p* = 0.40; Figure 1(b)). A similar effect was seen with regard to the liver to body weight ratio at time of sacrifice (3.0% in the MSC-CM group versus 2.9% in the UM group, *p* = 0.31; Figure 1(c)).

### 3.2. MSC-CM Treatment Provides Cytoprotective Effects

We investigated hepatic tissue injury 6 and 24 hours after IRI alone as well as 48 hours after IRI + PH by analyzing H&E stained liver tissue sections for signs of necrosis (accompanied by increased neutrophil infiltration). Sections were classified based on the percentage of necrotic tissue (no injury, <25%, 25–50%, 50–75%, or >75% of liver tissue affected).

**Figure 2:**
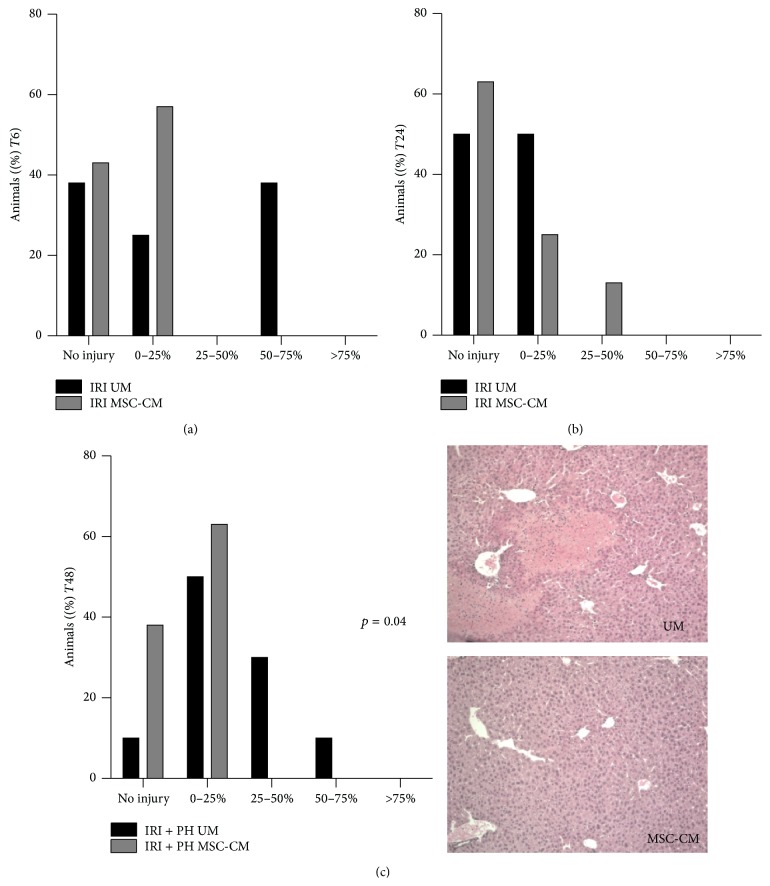
Effects of MSC-CM on hepatic tissue injury. H&E stained liver tissue sections were classified based on the percentage of damaged tissue: no injury, 0–25%, 25–50%, 50–75%, or >75% of liver tissue affected. This figure shows the percentage of animals with a certain injury score (a) 6 hours after IRI, (b) 24 hours after IRI, and (c) 48 hours after IRI + PH with representative pictures showing approximately 25% necrotic liver tissue in the UM group compared to no clear necrotic tissue in the MSC-CM group.

At 6 hours after IRI, no statistically significant difference in the percentage of necrotic tissue was found between the MSC-CM and UM treatment group, though a trend appeared toward reduced hepatic injury after MSC-CM treatment (MSC-CM versus UM treatment: no injury 43% versus 38%, <25% injury 57% versus 25%, and 50–75% injury 0% versus 38%; *p* = 0.18; Figure 2(a)). Similar results were found 24 hours after IRI (MSC-CM versus UM treatment: no injury 63% versus 50%, <25% injury 25% versus 50%, and 25–50% injury 13% versus 0%; *p* = 1.00; Figure 2(b)). After IRI + PH, however, MSC-CM treatment significantly decreased the percentage of necrotic tissue compared to UM treatment, with 38% versus 10% of animals showing no signs of injury, 63% versus 50% with <25% injury, and 0% versus 40% with more than 25% injury at 48 hours after surgery (*p* = 0.04; Figure 2(c)).

**Figure 3:**
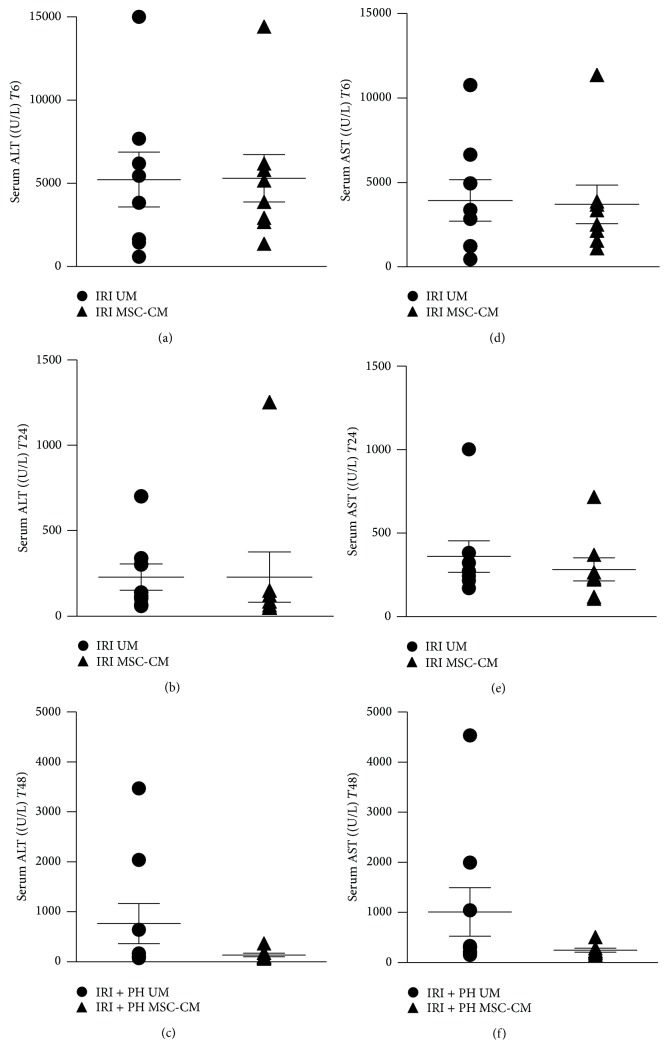
Effects of MSC-CM on serum injury markers. Serum ALT levels at (a) 6 hours after IRI, (b) 24 hours after IRI, and (c) 48 hours after IRI + PH. Serum AST levels at (d) 6 hours after IRI, (e) 24 hours after IRI, and (f) 48 hours after IRI + PH.

Additionally, serum transaminase levels were investigated as markers for hepatic injury. In the IRI alone group, serum ALT levels after 6 hours were 5301 ± 1426 IU/L in the MSC-CM treated group versus 5225 ± 1654 IU/L in the UM treated group (*p* = 1.00; Figure 3(a)). After 24 hours, ALT levels were reduced to 229 ± 147 IU/L after MSC-CM treatment versus 229 ± 77 IU/L after UM treatment (*p* = 0.23; Figure 3(b)). Similar results were found for AST levels (*p* = 1.00 at 6 hours and *p* = 0.33 at 24 hours, resp.; Figures 3(d) and 3(e)). In contrast, 48 hours after IRI + PH serum ALT and AST levels were markedly lower in the MSC-CM treated animals compared to the UM treated animals, though differences did not reach statistical significance (MSC-CM versus UM treatment: ALT 138 ± 35 IU/L versus 764 ± 399 IU/L, *p* = 0.18, and AST 248 ± 41 IU/L versus 1008 ± 484 IU/L, *p* = 0.14; Figures 3(c) and 3(f)).

### 3.3. MSC-CM Treatment Stimulates Hepatocyte Proliferation after IRI + PH

**Figure 4:**
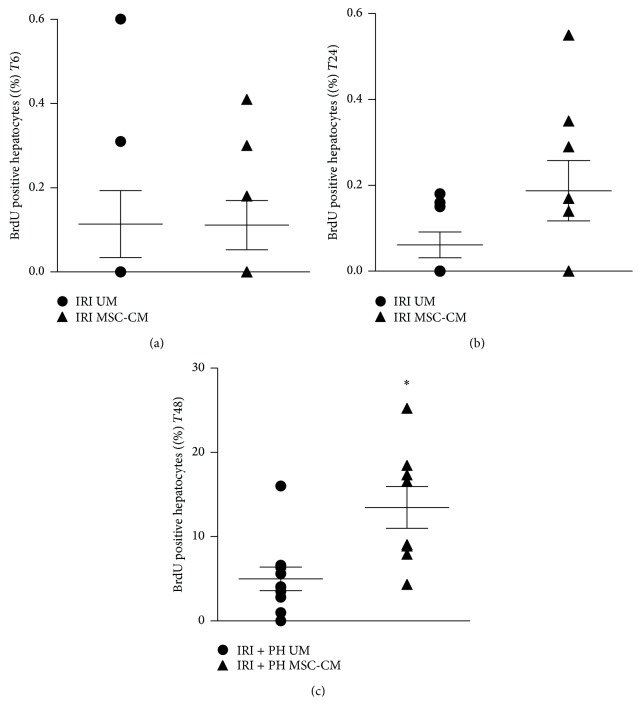
Effects of MSC-CM on hepatocyte proliferation. Livers were processed for immunohistochemistry on BrdU to quantify hepatocyte proliferation. This figure shows the percentage of BrdU positive hepatocytes (a) 6 hours after IRI, (b) 24 hours after IRI, and (c) 48 hours after IRI + PH; ^*∗*^ *p* ≤ 0.05.

After loss of liver mass, hepatocytes are triggered to enter the cell cycle and proliferate until tissue loss is compensated and homeostasis is restored, showing a proliferation peak in rodents around day 2 after liver tissue injury. Hepatocyte proliferation in the IRI alone group was not increased after 6 hours, independent of the treatment strategy (0.1% versus 0.1%, *p* = 0.85; Figure 4(a)). After 24 hours, MSC-CM treatment appeared to slightly induce hepatocyte proliferation, though proliferation levels were still low and showed no significant difference between treatment groups (0.19% after MSC-CM treatment versus 0.06% after UM treatment, *p* = 0.22; Figure 4(b)). In contrast, IRI + PH resulted in a clear increase in hepatocyte proliferation after 48 hours, with an almost 3-fold higher proliferation index in the MSC-CM treated animals compared to the UM treated animals (13.5% versus 5.0%, *p* = 0.002; Figure 4(c)).

### 3.4. Treatment with MSC-CM Does Not Significantly Affect Intrinsic Gene Expression Levels

Next, we investigated if treatment with MSC-derived factors affected hepatic expression levels of inflammation, proliferation, and angiogenesis related genes. Tumor necrosis factor alpha (TNF-*α*) and interleukin 6 (IL-6) are proinflammatory cytokines and crucial priming factors for hepatocytes to enter the cell cycle. Downstream in this process, the G1 to S phase transition is associated with upregulation of several cyclins including cyclin D, whereas transforming growth factor beta (TGF-*β*) is involved in the negative feedback on hepatocyte proliferation. Vascular endothelial growth factor A (VEGF-A), vascular endothelial growth factor receptors 1 and 2 (VEGF-R1 and -R2), and angiopoietin 1 (Ang-1) are relevant factors for the regeneration of damaged or lost vasculature. Furthermore, MSCs are described to have anti-inflammatory capacities with an important role for interleukin 10 (IL-10) and interleukin 1 receptor antagonist (IL-1Ra) [24, 25].

**Figure 5:**
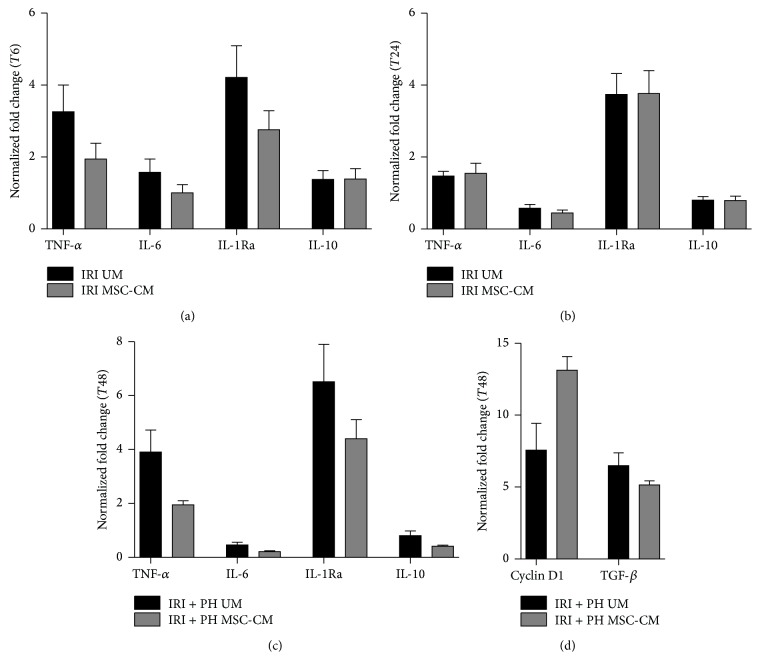
Effects of MSC-CM on hepatic gene expression. Hepatic gene expression levels were determined by quantitative RT-PCR and normalized against TBP. Expression levels of inflammation related genes at (a) 6 hours after IRI, (b) 24 hours after IRI, and (c) 48 hours after IRI + PH; (d) expression levels of cell cycle related genes at 48 hours after IRI + PH.

At 6 hours after IRI, MSC-CM treatment downregulated expression levels of the inflammatory genes* TNFA* and* IL1RN* compared to expression levels in the UM treated group (*TNFA* 40% reduction, *p* = 0.33;* IL1RN* 34% reduction, *p* = 0.51; Figure 5(a)), though results were not statistically significant. Similarly, in the IRI + PH model, downregulation of* TNFA* (50% reduction, *p* = 0.37) and* IL1RN* (33% reduction, *p* = 0.41) gene expression in the MSC-CM group was not statistically significant (Figure 5(c)). Furthermore, 48 hours after IRI + PH and MSC-CM treatment a trend toward upregulation of the cell proliferation stimulating gene* CCND1* (1.7-fold increase, *p* = 0.36) was seen versus a downward trend of the cell cycle inhibiting gene* TGFB* (21% reduction, *p* = 0.10; Figure 5(d)). None of the proangiogenic genes* VEGFA*,* FLT1*,* KDR*, and* ANGPT1* showed clear differences at any time point.

### 3.5. MSC-CM Contains Proteins Related to Hepatic Cell Proliferation

**Figure 6:**
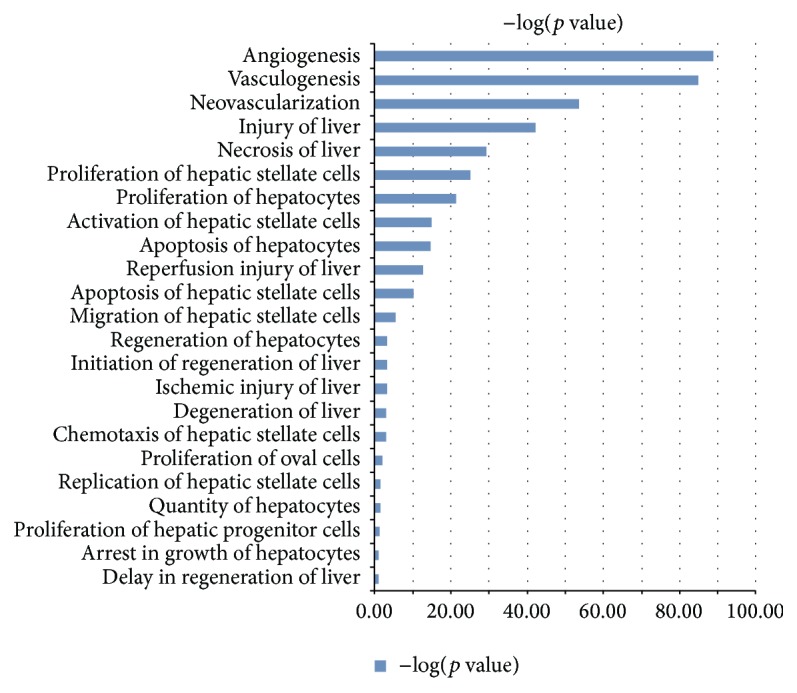
Functional analysis of cellular processes altered by L-MSC-conditioned medium. Proteins present in serum-free MSC-CM were analyzed using Mass Spectrometry. Out of 2060 proteins identified, 861 related to functional networks in Ingenuity Pathway Analysis. Top cellular processes involved proliferation of hepatocytes and nonparenchymal cells.

To elucidate the positive effect of MSC-CM on hepatic cell proliferation, but absence of amelioration of early IRI, we examined the secretome of the L-MSCs. In the conditioned medium, 2060 unique proteins were identified by Mass Spectrometry, of which 861 related to molecules in functional networks analyzed using Ingenuity Pathway Analysis. This functional network analysis showed a distinct pattern of cellular processes sustained by the MSC-CM: the top 10 processes were related to (induction of) cell replication and angiogenesis, rather than (attenuation of) inflammatory pathways. Proteins involved in hepatocyte and stellate cell proliferation, such as MAPK8, MAPK9, MAPK14, Akt, C3, C5, CAV1, and IL6, were enriched to a *p* value <3,56E-22 (Figure 6; complete table of components provided as Supplementary Table S1, in the Supplementary Material available online at http://dx.doi.org/10.1155/2015/202975).

## 4. Discussion

Hepatic ischemia and reperfusion (IRI) injury is a pathologic phenomenon that may occur in the situation of shock, severe liver trauma, liver resection under vascular occlusion, and (partial) liver transplantation. Subsequent elevated levels of reactive oxygen species (ROS) progress into oxidative stress, resulting in inflammation, damaged cellular components, and induction of apoptosis and necrosis of liver cells [26–30].

In recent years protective and regenerative effects of MSC therapy have been investigated in animal models of cerebral or myocardial infarction as well as after renal IRI [31–34]. Other reports describe the effectiveness of MSCs against toxic liver injury and hepatic failure [9–12, 20, 35]. MSCs appear to stimulate organ repair by affecting inflammation and inducing anti-apoptotic effects [24, 36–39]. They furthermore exert immunomodulatory effects on the immune response processes triggered during reperfusion [40].

Our group previously reported that trophic factors secreted by MSCs increase hepatocyte proliferation and alter expression levels of regeneration-related genes after partial hepatectomy, thereby stimulating liver regeneration [18]. The current study investigates MSC-secreted factors in a clinically relevant small-for-size ischemic liver model. Corresponding effects on hepatocyte proliferation were found with an almost 3-fold increase in BrdU positive cells, despite combined injury induced by 60 minutes of warm IRI and 50% hepatectomy. Recently, Du et al. described the use of bone marrow- (BM-) MSC-secreted factors for the first time in a 50% rat liver transplantation model [41]. In this elegant combined IRI and partial liver resection model, the cold and warm ischemic times were kept to a minimum (approximately 60 and 16 minutes, resp.), limiting the IRI component. Their results show similar effects with overall promoted liver regeneration. Moreover, our findings are in line with studies on injected MSCs in reduced size ischemic liver models, suggesting that proliferation stimulating effects are largely caused by paracrine interference of MSCs [42, 43].

Furthermore, a significant reduction in postischemic tissue injury after MSC-CM treatment was found in our combined injury model. This improvement in liver histology is also described in other small-for-size ischemic models using either injected MSCs or their secreted factors [41, 42, 44, 45]. Our histologic findings are supported by a clear decrease in serum transaminase levels in this group. MSCs thus seem to produce factors that help prevent oxidative stress to progress into cellular damage and necrosis.

Interesting however are the effects of MSC-CM early after IRI alone. Our results show no effects on serum transaminase levels and only a trend toward reduced tissue injury at 6 hours after reperfusion. In contrast, studies using bone marrow, adipose tissue, or umbilical cord derived MSCs in a liver IRI model have shown that treatment with these cells and/or their secreted factors significantly decreases serum ALT and AST levels [46–49]. An explanation for these different results could be the duration of ischemic injury, which was induced for 90 minutes in our model, whereas the models described in literature used 30 to max 60 minutes of ischemia. The oxidative damage induced by 90 minutes could be too severe to detect differences at early time points, that is, 6 and 24 hours after reperfusion. Another explanation may be found in the compounds present in the conditioned medium of L-MSCs. The identified proteins seem to fit into networks stimulating hepatocyte and stellate cell proliferation more prominent than attenuating inflammation or ischemic injury.

Though a trend was seen toward decreased TNF-*α* and IL-1Ra gene expression as well as increased cyclin D1 gene expression, the beneficial effects of MSC-CM treatment could not sufficiently be explained by changes in inflammation or proliferation related gene expression levels. This is in contrast with our previous findings on MSC-CM treatment in a clean partial hepatectomy model and might therefore be caused by interfering IRI-induced cascades. However, several other research groups using small-for-size ischemic liver models reported significant up- or downregulation of these inflammatory and/or proproliferative genes as early as 6 hours up to 72 hours after reperfusion [41, 45, 50]. In the current model we chose to investigate changes after 48 hours, according to the time point at which we found the most significant induction of proliferation in our previous study. Probably, changes in cytokine or chemokine gene expression preceded this peak in proliferation, which could explain the lack of significant gene expression changes at 48 hours.

Such could also explain the absence of significant gene expression changes of proangiogenic factors. A little light on this may further be shed by the recent publication of Hu et al., showing downregulation of angiopoietin 2 (Ang-2) in the early phase of liver regeneration (days 0–3) with recovery of Ang-2 levels during the later phase of liver regeneration (days 4–8) [51]. Complementary in vitro studies showed that the hepatoproliferative effect of Ang-2 is regulated through the decreased expression of TGF-*β*1. A different mechanism accounted for the stimulatory effects of Ang-2 on the later angiogenic phase of liver regeneration whereby Ang-2 upregulates VEGFR-2 through autocrine stimulation of the Ang-2 receptor, Tie2. As we determined Ang-1, we may have missed the effect through TFG-*β* during the early phase, whereas the indirect effect by induced proliferation of nonparenchymal cells through VEGF-R signaling probably takes 4-5 days to become evident. Another possible explanation could be that the substantial presence of angiogenesis related factors in MSC-CM largely meets the need for proangiogenic factors after injury and thereby inhibits upregulation of proangiogenic genes in the recipient. The exact mechanism of how MSC-secreted factors interfere with tissue injury and repair at a genomic and molecular level therefore needs to be further explored. Interestingly, the adenosine receptors ADORA2A and ADORA3, members of the G protein-coupled receptor (GPCR) family, were found to be present in the MSC-CM. These receptors are known for the preconditioning effect in myocardial ischemia and may play a role here as well.

Significant differences in liver weight reconstitution were not detected in our study. In contrast, Seki and coworkers, in their model of MSC treatment after combined IRI and 70% hepatectomy, describe an increase in regenerated liver weight as early as day 2 [50]. However, they induced only 15 minutes of ischemia compared to 60 minutes in our model, resulting in significantly lower oxidative stress related injury and thereby preserving more functional hepatocytes that can contribute to regeneration by proliferation. On the other hand, our findings are in line with those of Wang et al. and Kanazawa et al., showing a significant increase in liver weight not earlier than three or seven days after surgery, respectively [44, 45].

Kanazawa and coworkers described their experience with BM-MSCs in a model of 70% hepatectomy after 40 minutes of warm ischemia [44]. BM-MSCs were infused in the portal vein directly after liver resection, resulting in a decrease in liver tissue injury, including vacuolar changes and necrosis, and accelerated regeneration. However, as the authors discuss, the optimal route and dosage of MSC administration remain unclear. Systemically transplanted MSCs are mostly trapped in the microvasculature of the lung after intravenous infusion, because of their size and adhesion potential [52]. Direct injection into the portal vein, on the other hand, seems effective but might be unsafe. The number of MSCs needed for therapeutic effects is not known and ranges from 2 to 10 million MSCs per kilogram in small animal experiments, whereas fatal embolism has been described for injections exceeding 10 million cells overall [53]. Furthermore, concern has been raised on the possibility of malignant transformation of transplanted MSCs [54, 55]. The use of MSC-derived factors may therefore have important advantages over the use of MSCs. Potential other advantages are the elimination of the risk of rejection by the recipient's immune system, as well as the feasibility to produce the factors ready-to-use in large clinical grade quantities. Recent reports also suggest a similar beneficial effect of treatment with MSC-CM compared to treatment with MSCs [56, 57].

On the other hand, MSCs may be able to adjust to changing needs of the recipient, and much is still unclear about how different culture conditions affect the many factors present in MSC-CM. So far there is no consensus on a standardized method of production or on the optimal administration route or dosage of MSC-secreted factors. Furthermore, research on possible adverse effects is needed, such as overstimulation of inflammatory responses, hypersensitivity to certain compounds, or interference with metabolic processes. Another topic for future research should be to determine which (combination of) factors present in MSC-CM are responsible for the beneficial effects, to further optimize treatment strategies.

The current study was conducted to explore the effects of MSC-secreted factors on liver regeneration in the setting of reduced-size ischemic livers and to investigate if any beneficial effects could be clarified by effects on tissue damage after ischemia and reperfusion. We found that MSC-CM does promote liver regeneration, but not through amelioration of the IRI component. Though the exact mechanisms of MSC-mediated stimulation of tissue repair are still largely unclear, our results contribute to the increasing evidence suggesting an important role of paracrine effects by MSC-secreted trophic factors.

## 5. Conclusions

Our study confirms that MSC-secreted factors decrease hepatic tissue injury and promote liver regeneration after large liver resections, even in an ischemia and reperfusion injury environment. MSC-derived factors represent a promising strategy for safe and ready-to-use therapeutic intervention to stimulate organ repair and regeneration in the setting of small-for-size syndrome and postresectional liver failure. However, the optimal source of MSCs for this conditioned medium as well as the dosage still needs to be elucidated.

## Supplementary Material

Supplementary Table S1: To elucidate effects of MSC-CM on ischemia and reperfusion injury and hepatic proliferation, proteins present in the twenty-five-fold concentrated serum-free MSC-CM (50-kD molecular cut-off filters) was analyzed by Mass Spectrometry.

## Figures and Tables

**Table 1:** RT-PCR primer sequences.

Gene	Name	Accession number	Primer (forward/reverse)
*TNFA *	Tumor necrosis factor alpha	NM_013693	CCCTCACACTCAGATCATCTTCT GCTACGACGTGGGCTACAG

*IL6 *	Interleukin 6	NM_031168	TAGTCCTTCCTACCCCAATTTCC TTGGTCCTTAGCCACTCCTTC

*IL1RN *	Interleukin 1 receptor antagonist	NM_031167	GCTCATTGCTGGGTACTTACAA CCAGACTTGGCACAAGACAGG

*IL10 *	Interleukin 10	NM_010548	GCTCTTACTGACTGGCATGAG CGCAGCTCTAGGAGCATGTG

*CCND1 *	Cyclin D1	NM_007631	GCGTACCCTGACACCAATCTC CTCCTCTTCGCACTTCTGCTC

*TGFB *	Transforming growth factor beta	NM_011577	CTCCCGTGGCTTCTAGTGC GCCTTAGTTTGGACAGGATCTG

*KDR *	Vascular endothelial growth factor receptor 2	NM_010612	TTTGGCAAATACAACCCTTCAGA GCAGAAGATACTGTCACCACC

*ANGPT1 *	Angiopoietin 1	NM_009640	CACATAGGGTGCAGCAACCA CGTCGTGTTCTGGAAGAATGA

*VEGFA *	Vascular endothelial growth factor A	NM_009505	GCACATAGAGAGAATGAGCTTCC CTCCGCTCTGAACAAGGCT

*FLT1 *	Vascular endothelial growth factor receptor 1	NM_010228	TGGCTCTACGACCTTAGACTG CAGGTTTGACTTGTCTGAGGTT

*TBP *	TATA binding protein	NM_013684	AGAACAATCCAGACTAGCAGCA GGGAACTTCACATCACAGCTC
